# Distinction of Microglia and Macrophages in Glioblastoma: Close Relatives, Different Tasks?

**DOI:** 10.3390/ijms22010194

**Published:** 2020-12-27

**Authors:** Susan Brandenburg, Anne Blank, Alexander D. Bungert, Peter Vajkoczy

**Affiliations:** 1 Department of Experimental Neurosurgery, Charité—Universitätsmedizin Berlin, Corporate Member of Freie Universität Berlin, Humboldt-Universität zu Berlin, and Berlin Institute of Health, 10117 Berlin, Germany; susan.brandenburg@charite.de (S.B.); anne.blank@charite.de (A.B.); alexander.bungert@charite.de (A.D.B.); 2 Department of Neurosurgery, Charité—Universitätsmedizin Berlin, Corporate Member of Freie Universität Berlin, Humboldt-Universität zu Berlin, and Berlin Institute of Health, 10117 Berlin, Germany

**Keywords:** myeloid cells, glioma, tumor microenvironment

## Abstract

For decades, it has been known that the tumor microenvironment is significant for glioma progression, namely the infiltration of myeloid cells like microglia and macrophages. Hence, these cell types and their specific tasks in tumor progression are subject to ongoing research. However, the distribution of the brain resident microglia and the peripheral macrophages within the tumor tissue and their functional activity are highly debated. Results depend on the method used to discriminate between microglia and macrophages, whereby this specification is already difficult due to limited options to distinguish between these both cell populations that show mostly the same surface markers and morphology. Moreover, there are indications about various functions of microglia and macrophages but again varying on the method of discrimination. In our review, we summarize the current literature to determine which methods have been applied to differentiate the brain resident microglia from tumor-infiltrated macrophages. Furthermore, we compiled data about the proportion of microglia and macrophages in glioma tissues and ascertained if pro- or anti-tumoral effects could be allocated to one or the other myeloid cell population. Recent research made tremendous efforts to distinguish microglia from recruited macrophages. For future studies, it could be essential to verify which role these cells play in brain tumor pathology to proceed with novel immunotherapeutic strategies.

## 1. Introduction

Glioblastomas belong to primary human brain tumors of WHO grade IV [1] with highest malignancy and aggressiveness. These tumors represent up to 60–70% of all malignant gliomas, have a poor clinical outcome and high recurrence rates [2]. Despite therapeutic and diagnostic efforts in the last decades, glioblastoma is still a cancer with a five-year survival rate of only ~5% [3,4,5,6]. Thus, a better understanding of glioma biology is required, and new and effective therapeutic strategies must be developed.

The treatment of glioblastomas is a difficult task due to its complex cell composition. A special feature of glioblastomas are the high infiltration rate of myeloid cells whereby the resident immune cells of the central nervous system (CNS), microglia, as well as peripheral macrophages belong to the tumor tissue [7,8,9] and make up to 30–50% of the total tumor mass [10]. In the healthy CNS, microglia continuously scan their environment. These cells are important for surveying the structural and functional integrity of the CNS and for detecting of disturbances of homeostasis. In response to pathological stimuli, they are activated and turned into phagocytic, antigen-presenting, and lymphocyte activating cells. In the tumor context, glioma cells secrete chemokines and growth factors, which attract microglia and macrophages, leading to their accumulation within the tumor area [11,12]. Data regarding the function of microglia/macrophages in glioma remain controversial. Latest findings elucidate that in glioma accumulated microglia/macrophages are a mixed cell population with pro- and anti-tumoral properties [13,14,15]. However, tumor-supporting effects of myeloid cells seem to predominate [16,17,18,19] even if anti-tumoral features were reported [14,20]. Commonly, microglia and macrophages are named tumor-associated macrophages (TAMs) and are analyzed as one cell population [21,22,23,24], implicating that both cell fractions have the same function in the tumor area. However, there is evidence that microglia and macrophages behave differently and could have various functions in glioma [25,26]. Thus, a clear discrimination of microglia and macrophages could have advantages for the development of future therapeutic approaches in glioblastoma treatment.

## 2. Origin and General Function of Microglia

The ontological origin of microglia was intensively debated [27], but current publications make clear that the brain-resident microglia derived from progenitors of the yolk sac [28,29]. It was demonstrated that microglia progenitor cells enter the CNS before day 8 of the embryonal development in mice (E8) [28] and are present in the cephalic mesenchyme and neuroepithelium from E10.5. For human microglia, three main routes of CNS infiltration were discovered [30,31]: (i) Starting from 4.5–5.5 gestation weeks (gw), a penetration of amoeboid microglia precursor cells could be observed in the forebrain. (ii) While the migration continued from meninges to parenchyma bordering the diencephalic–telencephalic fissure, cells entering the CNS from the choroid plexus moved in direction to the eminentia thalami. (iii) Furthermore, ventricles were identified as entrance route. From 8 gw, microglia infiltrated the cortical plate and accumulated in ventricular and intermediate zones, presubplate, inner cortical plate and marginal zone. The cluster building was observed in or near white-matter tracts followed by proliferation in 8–12 gw, while proliferation started earlier in the preplate of the cerebral wall (gw 5.5) as well as in the intermediate marginal zone (7–8 gw). Depending on the localization, morphology, and distribution of microglia changes during embryogenic development, from gw 9 the ramified phenotype was predominant throughout the brain parenchyma and became evenly distributed. These changes were acquired later (19 gw) in the cortex.

The microglia represent a long-living cell population that has self-renewal capability [28,32,33] Interestingly, besides yolk sac-derived microglia, perivascular, meningeal, and choroid plexus macrophages are present in the brain. These macrophages are replaced by fetal liver-derived progenitor cells that are part of common hematopoiesis [32,33,34]. In addition, if a pathological stimulus occurs, circulating monocytes, derived from hematopoietic stem cells and matured in the bone marrow [35], can migrate to the CNS, where these cells differentiate into tissue macrophages, implicating high diversity of myeloid cell populations within the diseased brain.

The brain is an immune privileged organ, which is protected by the blood–brain barrier (BBB) that impedes the entry of circulating immune cells to the CNS under physiological conditions [36,37]. Thus, especially the brain-resident microglia are important for brain tissue homeostasis and immune defense [38,39,40]. Under homeostasis, microglia continuously sensing their surroundings to detect disturbances [41]. Microglia respond to injury and infection [42,43], but are also relevant for CNS development and function, e.g., neurogenesis and myelinogenesis [44,45]. In almost all brain diseases microglia are involved in inflammatory reactions, amongst others, in traumatic brain injury [46] and CNS tumors [47].

## 3. Distinction of Microglia and Macrophages in Glioma

High amounts of myeloid cells infiltrate the tissue of human and murine glioblastomas [7,8], whereby a direct correlation between the grade of malignancy and the number of myeloid cells was demonstrated [7]. Here, the myeloid cells consist of brain-resident microglia and infiltrated macrophages from the blood stream, which were often considered cumulatively by using the term “tumor-associated macrophages” (TAMs) or “glioma-associated macrophages” (GAMs). TAMs were identified as being highly relevant for tumor progression, responsible for resistance to anti-angiogenic treatment approaches [48,49,50,51] and play an important role in immune escape mechanisms [52], therefore TAMs were taken into consideration as therapeutic targets in glioblastoma [53,54,55]. Hence, these cells are of particular importance for glioma research and development of novel therapeutic strategies. However, so far, it is not clarified if a joint examination of TAMs is appreciated or a more detailed analysis with separately looking at microglia and macrophages is preferable to initiate more target-specific therapies. Despite the different origin of microglia and macrophages, these cells are hard to differentiate due to a joint expression of surface markers, including IBA1, CD11b, CD45, F4/80, CD68, and CX3CR1 [56,57,58]. Various strategies for the distinction of microglia and macrophages were established and have been applied in glioma animal models and partly to human specimen. Furthermore, novel approaches for myeloid cell differentiation were developed, predominantly using gene sequencing [59,60,61].

### 3.1. Classical Differentiation by CD45 Expression Level

**Figure 1:**
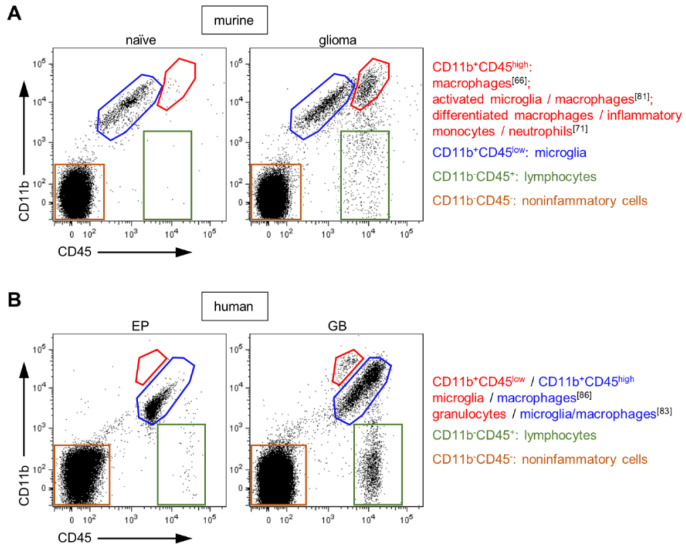
Flow cytometry analyses of brain tissues by CD11b and CD45 staining. (**A**) Depicted are dot plots of a naïve mouse brain and a glioma-bearing (GL261) hemisphere (adapted from Brandenburg et al. [15]). Dead cells were excluded for analyses by DAPI^+^ staining. In all publications using rodent glioma models, the CD11b^+^CD45^low^ population is described as microglia. In contrast, the CD11b^+^CD45^high^ population is referred to different cell fractions as indicated. *Blue*-marked cell fraction: CD11b^+^CD45^+^ population present in naïve and tumor-bearing brains. (**B**) Dot plots of human brain tissues from an epilepsy patient (EP) and a patient suffered from glioblastoma (GB) are presented (adapted from Blank et al. [81]). DAPI^+^ cells were excluded from analyses. For humans, data are not consistent about composition of the entire CD11b^+^CD45^+^ population. *Blue*-marked cell fraction: CD11b^+^CD45^+^ population present in EP and GB specimens, implicating a contribution of microglia.

A favored experimental approach to differentiate between microglia and macrophages in glioblastoma is their expression level of CD45. CD45 is a protein tyrosine phosphatase that is expressed on all nucleated hematopoietic cells and relevant for antigen receptor signal transduction, development of lymphocytes [62] and for macrophage-mediated adhesion [63]. Microglia were defined as CD45^low^ and macrophages as CD45^high^ expressing cells [64], accordingly discrimination is solely possible by flow cytometry (Figure 1A) and not by histology. This concept is well accepted and has been used in many rodent glioma studies [65,66,67]. Badie and Schartner [64] based this classification on Sedgwick et al., where total body irradiated chimeric rats with inflamed CNS were analyzed [68]. Here, the CD45^low^ population solely originated of the recipient and accordingly referred to microglia, while the CD45^high^ population was exclusively determined as derived from the donor indicating the infiltrating macrophages. Some studies go even further where the CD45^high^ population was additionally subdivided into inflammatory monocytes recently infiltrated from the blood circulation and differentiated tissue macrophages by expression of Ly6C^high^ or Ly6c^low^, respectively [69].

It is assumed that microglia are not able to upregulate CD45, implicating a stable CD45 expression what seems to be true under physiological conditions. However, for the diseased CNS the CD45 levels of microglia are a matter of controversy. There many reports exist about upregulation of CD45 by microglia in vitro and in vivo under certain pathological conditions. Here, the capacity of microglia to increase CD45 depends on disease [70,71,72,73] and CNS area. For example, microglia of the retina preserve a CD45^low^ phenotype in a light injury model and could be discriminated from infiltrating macrophages [74], while microglia of the spinal cord increased CD45 expression in a demyelination model [75]. Notably, it was demonstrated that myeloid cells showed increased expression and activity of CD45 if cells were exposed to hypoxia [76] while hypoxia is a hallmark of glioma [77,78]. Especially in the brain tumor context, our group clearly demonstrated a contribution of microglia to the CD45^high^ population in vivo, if gliomas in chimeric mice generated by head-protected irradiation were investigated [79]. Moreover, using the busulfan model to generate chimerism elucidates that the occurrence of microglia is not only restricted to the CD45^low^ fraction but also can be found in the CD45^high^ population in glioma tissues [80]. Our implemented in vitro assays confirmed the observation that microglia have the capability to upregulate CD45. We could show that isolated microglia cells from adult mouse brains increased their CD45 expression levels following cultivation with tumor-conditioned medium [79]. Co-culture of microglia and granulocytes under tumor conditions even accelerated the CD45 expression on microglia [81]. Data implicate that the CD45^low^ population may represent the non or little activated microglia in the tumor hemisphere while the CD45^high^ cells could be referred as glioma-activated cells composed of microglia and macrophages [15,79]. Consequently, CD45 appears to be less sensitive for discriminating the activated microglia population from tumor-infiltrating macrophages in gliomas.

Furthermore, attempts were made to assign this concept to human glioblastoma, but early on it was discussed whether the CD45 expression level is really an appreciate parameter for microglia and macrophages in human glioblastoma tissue [10,54,82]. It was described that myeloid cells upregulate CD45 in human glioblastoma samples, but myeloid subpopulations were not differentiated [83]. However, Parney at al. defined two CD11b^+^ cell populations in cell suspensions of human glioblastoma samples: CD11b^+^CD45^high^ and CD11b^+^CD45^low^ and referred to them, in analogy to the murine model, as macrophages and microglia [84]. Here, the CD11b^+^CD45^low^ population was not further characterized and no nontumor tissue was used as control. Recently, we determined that the CD45^low^-expressing cell population in human glioblastoma tissue resembles rather granulocytes, as proven by staining of the neutrophil marker CD66b [81]. This implicates that the CD45 expression level is inadequate for the discrimination of microglia and macrophages in human glioblastoma specimens (Figure 1B).

Consequently, while analyzing the CD45 expression by flow cytometry in order to distinguish between microglia and macrophages is a simple method, and thus widely used, new evidence has shown that this technique is insufficient in glioma rodent models as well as in human specimens.

### 3.2. Bone Marrow Chimeras Generated by Different Irradiation Strategies

Another method to distinguish between microglia and macrophages is the generation of bone marrow chimeras [82]. Here, mice were irradiated, which causes elimination of stem cells as well as progenitor cells. Afterwards, labeled bone marrow cells from donor animals will be transplanted. The labeling allows the distinction of circulating immune cells from radio-resistant microglia [85]. Recently, the total body irradiation (TBI) has been challenged due to potential adverse effects on CNS homeostasis [86,87] based on alterations in the blood–brain barrier integrity which could be responsible for unspecific influx of cells from the circulation into the brain [79,88]. Here, up to 18% of the entire CD11b^+^CD45^+^ myeloid cell population can belong to the infiltrated macrophages without specific pathological stimulus [79]. Thus, new methods such as the parabiosis model [89], and irradiation with a head shield [79,90] were established in order to circumvent changes of the BBB. The parabiosis technique, where two organisms temporarily joined their circulatory systems, is not allowed in each country, and showed relatively low frequencies of donor-derived immune cells in the blood of target mice, precluding a complete detection of infiltrated cells within the brain. In contrast, our strategy using head-protected irradiation (HPI) led to high reconstitution levels of monocytes in the blood, avoids false-high infiltration rates in inflamed tissues and experimental artifacts as observed after TBI in a glioma mouse model [79] and in a model of spinal cord injury [91]. Furthermore, using this model we could show that macrophages infiltrate only at the late stage of tumor growth (d21), and microglia contribute to 40% of the CD11b^+^CD45^high^ fraction of murine glioma-bearing hemispheres [79], again questioning the primary classification by the CD45 expression level. Nonetheless, depletion using head protection has the limitations that thymus-like structures localized in the neck would be also shielded by the helmet and may lead to lower reconstitution efficiency [79,92]. Additionally, irradiation resulted in little immune cell activation in the periphery that could influence the migratory behavior of peripheral monocytes [80].

### 3.3. Chimeric Mice Generated by Busulfan Administration

Besides irradiation strategies, myeloablative chemotherapy by application of busulfan followed by transfer of labeled bone marrow cells can be used to generate chimeras [93,94,95]. Busulfan leads to effective myeloablation in the blood [94] and busulfan-treated chimeras revealed same reconstitution levels as described for TBI chimeras [93,96]. However, greater concentrations of busulfan resulted in a higher influx of labeled macrophages into the brain compared to total body irradiated animals [93], implicating an additional increase of unspecific infiltration of circulating donor-derived cells passing the BBB following busulfan application, although a preserved BBB integrity was described [95,96]. Thus, busulfan-induced myeloablation leads to chimeric mice and presents a possibility to discriminate between brain-resident microglia and labeled infiltrating macrophages but with side effects, which do not correspond to the physiological condition in the naïve brain. Nevertheless, recently low dose busulfan (stated as nonmyeloablative) was applied to study the microenvironment and biology of gliomas [80,96]. HPI and nonmyeloablative strategy showed less unspecific macrophage influx to contralateral hemispheres than pre-treatment with high dosage of busulfan [79,96]. The direct comparison of different busulfan application and head protected irradiation confirmed these observations [80].

### 3.4. Reporter Mice Based on the Cx3cr1 Gene

Recently, reporter mice were used to differentiate between microglia and macrophages in glioma models. The fractalkine receptor gene *Cx3cr1* is the basis of several mouse lines. The *Cx3cr1-eGFP* mice (homo- or heterozygous) express GFP under the control of the *Cx3cr1*-promoter [97]. Under homoeostasis the microglia express high levels of CX3CR1. Thus, microglia are markedly positive for GFP while peripheral monocytes showed less GFP expression leading to their classification that microglia in the glioma tissue are CX3CR1^high^ and macrophages show a CX3CR1^low^ phenotype [98]. Notably, it is not clear until now, if microglia and macrophages could adjust their CX3CR1 expression when infiltrating the tumor tissue. For instance, bone marrow cells of *Cx3cr1^GFP/WT^* mice were used to generate chimeras for the detection of infiltrated macrophages in brain tumors by their GFP signal demonstrating pronounced CX3CR1 expression of glioma-associated macrophages [69,99]. Moreover, these mice were used to image both microglia and infiltrated macrophages by in vivo imaging of glioblastoma tissues [100,101]. Therefore, using CX3CR1 as a microglia marker is reasonable under physiological conditions but a strong pathological stimulus like glioma progression could affect expression and should be further examined. Another interesting reporter mouse strain is the *Cx3cr1^GFP/+^Ccr2^RFP/+^* mouse [102]. The usage is based on the assumption that microglia express CX3CR1 but no CCR2 while monocytes/macrophages show CCR2 expression but only low levels of CX3CR1 [103]. This mouse allowed the visualization of microglia and macrophages, since microglia could be depicted as green fluorescent and macrophages express the red fluorescent protein and is often applied in glioma rodent models [71,104,105]. However, it should be mentioned that expression of CCR2 on microglia (CD11b^+^CD45^low^ cells) was described recently [106,107], wherefore using this mouse line must be reconsidered. Additionally, a tamoxifen inducible *Cx3cr1^CreER^* mouse was crossed with a R26^Reporter^ mouse. CreER is constitutively expressed under the control of the *Cx3cr1*-promoter, while R26^Reporter^ is only expressed following Cre recombination activated by tamoxifen. This yielded in CX3CR1^+^R26^Reporter+^ cells including microglia and macrophages. However, Cre activation is limited. Consequently, several weeks following tamoxifen application, monocytes of the circulation lose the R26^Reporter^ expression while R26^Reporter^ labeling is almost permanent in long-lived microglia [74,104], demonstrating a sophisticated method to discriminate between microglia and macrophages that should be applied in the glioma model.

### 3.5. Novel Markers of Microglia

Recently, analyses were performed to identify specific marker for microglia which would be the basis for an adequate differentiation between microglia and macrophages. In this context signature genes of microglia of the homoeostatic brain were identified, including *Tmem119*, *Sall1*, *P2ry12*, *Olfml3*, *Hexb*, *Fcrls*, *Siglech*, *Tgfbr1,* and *Gpr34* [60,105,108,109,110,111,112]. A part of these genes was used to develop new reporter mice to label and/or genetically manipulate the microglia cell population [113]: *Tmem119^eGFP^* and *Tmem119^CreERT2^* [114], *Tmem119^TdTomato^* [115], *Sall1^GFP^*, and *Sall1^CreER^* [116,117], and *Hexb^TdTomato^* and *Hexb^CreERT2^* [118], and *P2ry12^CreER^* [119].

In general, all these new genes were determined as microglia specific by using the CD11b^+^CD45^low^ cell population of the naïve brain [60,105,108,109] in comparison to other CNS cell types, which include astrocytes, neurons, and oligodendrocytes [60,109], other tissue macrophages [105], or peritoneal macrophages [108]. Thus, microglia were not compared to brain-infiltrated macrophages therefore limiting the statement about marker specificity. Notably, several of these microglia genes such as *Tmem119*, *Sall1,* and *P2ry12* could be downregulated in different brain pathologies [120,121,122] and are not completely restricted to the microglia cell population [118,119,123,124], which could additionally impede a distinct discrimination of microglia and macrophages in the diseased brain. *Hexb* showed the most stable expression also in the diseased brain (e.g., neurodegeneration), implicating that the *Hexb*^TdTomato^ reporter mice [118] should be considered for investigation of the myeloid cell composition of CNS tumors.

Additionally, to the gene signature approach, Bowman et al. also postulated CD49D as a marker for discrimination whereby microglia should be negative for CD49D and macrophages showed an CD49 expression [59]. This marker was used for flow cytometric analyses to differentiate between microglia and macrophages in glioma mouse models and human glioblastoma specimens [59,123,125,126]. However, to define the specific expression of CD49D within macrophages, microglia of naïve brains were compared to macrophages from spleen, liver, lung and bone marrow. Furthermore, in glioma, again the CD45 expression level provided the basis for microglia and macrophage distinction revealing CD45^low^ cells expressing negligible or no CD49D, whereas CD45^high^ cells showed high CD49D expression [59].

### 3.6. Depletion Strategies to Verify the Function of Microglia and Macrophages

Besides reporter mice, other techniques to study the function of microglia and macrophages were developed, using strategies to deplete one or the other myeloid cell population. This should allow the evaluation of the removed and remaining cells according their contribution and behavior. CSF1R inhibitors are postulated to be effective for depletion of microglia based on their dependency on CSF1R for survival [127]. Small molecules that can cross the blood-brain barrier were used as CSF1R inhibitors, e.g., PLX3397 and PLX5662, which showed high potency [127] and high brain penetrance [124], respectively. These inhibitors are effective to realize long-term depletion of microglia in vivo, but this method does not maintain the elimination of all microglia cells over time [128] and repopulation occurred [129]. Moreover, CSF1R inhibitors also could target peripheral immune cells including macrophages [130], and are able to change polarization status of macrophages from an immunosuppressive to proinflammatory phenotype [131]. Thus, the results of CSF1R inhibition must be considered carefully.

In *CD11b-HSVTK* transgenic mice, the expression of the suicide gene *HSVTK* is driven by the CD11b-promoter that is expressed by microglia and monocytes/macrophages. Here, apoptosis within CD11b positive cells is induced following application of ganciclovir (GCV). A systemic treatment with GCV should lead primarily to depletion of circulating monocytes and resulted in 45% less TAMs in the glioma tissue [20]. To allow the investigation of specific depletion of microglia, we and others established bone marrow chimeras using *CD11b-HSVTK* mice as recipients and donor-derived labeled bone marrow cells [26,132]. Thus, HSVTK expression was restricted to the brain-resident microglia cells. Here, subsequent local intracerebroventricular infusion of ganciclovir enabled high level of microglia depletion of around 90%. However, depletion was not long lasting due to fast repopulation of the microglia cells, indicating a time frame of about two weeks for experimental setups [133]. Thus, this method is complex but suitable for short time investigations [26], including glioma mouse models whereby the remaining microglia cells should also be considered.

## 4. Function of Microglia and Macrophages in Glioma

Microglia and macrophages have different origins, but their functions appear similar at a first sight. Even if the number of myeloid cells in glioma was correlated with malignancy [7], data regarding their function were discussed controversially. Recent studies suggested an overexpression of both pro-inflammatory and immunosuppressive molecules by tumor-associated microglia/macrophages [19,20,130,134,135] indicating a mixed cell population different from defined M1 and M2 phenotype. However, tumor-supportive properties of microglia/macrophages seemed to predominate [16,17,18,19], although anti-tumoral effects were also described [14,20]. For instance, pharmacological activation of TAMs led to increased tumor volumes [16], while local depletion of myeloid cells resulted in strong reduced glioma progression [16,26]. Alternatively, depletion of primarily monocytes/macrophages by systemic ganciclovir application of *CD11b-HSVTK* mice enhanced tumor growth [20]. Interestingly, the treatment with several inhibitors that interfere with glioma physiology led to a switch of the immunosuppressive and pro-tumoral phenotype of microglia/macrophages to a rather anti-tumoral status associated with tumor regression [136,137,138,139], elucidating the relevance of the myeloid cells for therapeutic approaches in glioblastoma. Frequently, microglia and macrophages were not distinguished, but there are some indications about distinct functions.

In vitro assays using cell cultures showed that microglia from adult brains and bone-marrow-derived macrophages (BMDM) were able to upregulate PDL1 following stimulation with IFNγ but the expression level was much higher in BMDMs [140]. PDL1 is an immune inhibitory receptor ligand for the PD-1 receptor. Their binding leads to T cell dysfunction and apoptosis, and therefore facilitates the immunosuppressive microenvironment and progression of glioma. Consequently, both myeloid cell populations could be involved in this signaling. Furthermore, if microglia and macrophages were stimulated in vitro with tumor-conditioned medium, both cell populations increased CCL2 expression [106], which is relevant for recruitment of myeloid cells and regulatory T cells. Again, the induced molecule expression was higher in BMDMs than in the microglia fraction. Besides these inductions of different molecule expression, co-culture of microglia or BMDM with tumor cells revealed an enhancement of glioma cell invasion using both human and murine cells [134]. Thus, microglia and macrophages have the capacity to support invasive tumor cell behavior.

In an ex vivo glioma model using organotypic brain slices, bone marrow-derived macrophages upregulated pro-inflammatory cytokines such as *Il1a* and *Il1b* if co-cultured with organotypic tumor slices. In contrast, the microglia, isolated from postnatal pups, cultured with tumor slices showed no increase of these genes [135] implicating diverse cytokine expression following stimulation by tumor conditions.

In vivo it was observed, if total body irradiated chimeras with either labeled microglia or bone marrow-derived macrophages were analyzed, different morphologies of microglia and macrophages were found by intravital 2-photon microscopy. Bone marrow-derived macrophages showed small cell bodies with few branches and had a high migratory activity in glioma tissue while microglia were bigger, highly branched and rather stationary but sensing their microenvironment through the continuous extension and retraction of ramified processes [99]. These observations could evidence different migratory behavior of the myeloid cell populations. Furthermore, our group generated chimeras by head protected irradiation, *CD11b-HSVTK* mice were used as recipients for GFP^+^ labeled bone marrow cells and treated intraventricularly with ganciclovir to solely deplete the microglia. In this experimental setup, we could show a decrease in vessel density and slowdown in glioma growth indicating microglia as a crucial cell population in the regulation of tumor angiogenesis [26]. Sole macrophages were not depleted in this context to verify if the observed angiogenic function is really restricted to the microglia population [26]. Additionally, *Arg1* expression, a gene defining an anti-inflammatory phenotype, was analyzed in myeloid cells by separating them via analyzing CD45 expression level one week after tumor cell inoculation [106]. As already observed for other molecules in vitro, both cell populations expressed this gene but microglia (CD45^low^) to a lower extent as the macrophages (CD45^high^). In contrast, CCL2 was expressed by microglia and macrophages at comparable levels [106]. Interestingly, a study using immune checkpoint inhibitors in human glioblastoma and a glioma mouse model postulated that especially macrophages expressing high level of CD73 in the tumor are resistant to therapy and responsible for ongoing immunosuppression during therapeutic treatment [141].

Using gene signatures, attempts to differentiate microglia from infiltrating macrophages were made. Here, a predominance of blood-derived macrophages in upregulation of immunosuppressive cytokines and phagocytosis-related genes was described [125] based on the gene signature determined by Bowman et al. [59]. A study by Chen et al. reported the upregulation of proinflammatory cytokines and genes related to metabolism in microglia, while pathways associated to migration were a common characteristic of both cell populations but being the most regulated pathway category in macrophages [69], whereby microglia and macrophages were sorted by their expression profile CX3CR1^high^CCR2^-^ and CX3CR1^+^CCR2^+^, respectively. A common finding of several studies is a different localization pattern of the myeloid cell populations. Microglia were detected in the peripheral tumor area, while blood-derived macrophages were associated to blood vessels and necrotic areas especially in the tumor core. Here, defining microglia and macrophages by gene signature [59], determined that accordingly to location of the myeloid cells, their expression profile changed between periphery and tumor core [142]. Moreover, using the transcriptomic data [59], a microglia-restricted mTOR-related immunosuppression through STAT3 and NFκB pathways of microglia with promoted tumor progression was observed in glioma mouse models, while macrophages were noninvolved [143].

In summary, in vitro stimulated microglia and bone marrow-derived macrophages showed overexpression of immunosuppressive relevant proteins, but microglia expressed molecules less strong than the BMDMs. The ex vivo culture model implicates differences in activation of both cell populations. In vivo the function of microglia and macrophages remain questionable due to only few reports and the difficulty to compare the various methods of distinction. Thus, present data do not allow assigning a specific function to the microglia or macrophage population.

## 5. Proportion of Microglia and Macrophages in Glioblastoma

If the functions of microglia and macrophages differ, the question is whether microglia or macrophages predominate the glioma tissue. This would be an important aspect for development of future therapeutic strategies. Interestingly, reports about the composition of the myeloid cell population in gliomas are limited. However, based on the applied model, in literature different ratios of microglia and macrophages that infiltrate glioma tissues can be found (Table 1).

Badie and Schartner were the first to use the discrimination of microglia and macrophages by their CD45 expression level in various models of rat glioma, where allogeneic and syngeneic tumor cell grafts, such as C6, 9L and RG-2, were used [64]. They calculated the infiltration of the myeloid cell populations in relation to all living cells. Thus, the infiltration rates of both microglia and macrophages were relatively low. In all models, between 15% and 34% microglia (CD45^low^) and only 5–12% macrophages (CD45^high^) were observed. The RG-2 model showed the lowest frequencies of myeloid cells but in each tumor the microglia population prevailed [64]. This was confirmed by a recent study, where C6 glioma cells were implanted into rat brains. Here, it was found that the CD45^high^ population increased with tumor progression implicating a stronger influx of macrophages at a later time point of growth [66]. In a glioma mouse model (GL261), the same kinetic could be detected. Nevertheless, a higher proportion of infiltrating macrophages was observed and made up to 42% of the myeloid cell population at day 15 of progression [144]. Consequently, when discrimination is based on CD45 expression level of myeloid cells, more microglia occurred in glioma-bearing brains during early tumor progression but at later times macrophages gained a bigger share.

If chimeras were used to investigate the proportions of microglia and macrophages in glioma tissues, the observations varied. In total body irradiated mice, macrophages infiltrated early the brain tumor tissue (d7) and increased up to 65% on day 21 in relation to the entirely accumulated myeloid cell population whereby macrophages represented almost the complete CD45^high^ fraction [79]. Thus, in these chimeras, macrophages are the main population of myeloid cells in the brain tumor hemisphere. Nevertheless, using the head protected irradiation, our group demonstrated only a moderate influx of macrophages into glioma tissues not before day 21. Only 25% of the entire myeloid cells were macrophages on d21 and the macrophages constitute 65% of the CD45^high^ population implicating a contribution of up to 35% microglia to this cell fraction. In contrast to TBI where macrophages were detected additionally in the peritumoral area, the HPI method led to restriction of macrophages to the glioma mass [79]. All in all, HPI led to reduced influx of macrophages compared to TBI and microglia cells dominated the glioma brain hemisphere.

Results of chimeras generated by busulfan administration depended on the dosage of this chemotherapeutic agent. Frequently, the infiltration rate of macrophages was determined by flow cytometric analyses, whereby the tumor-bearing hemispheres were used, including non-tumor tissue with uninitiated microglia. This could lead to an assumption of false-high distribution of microglia to the tumor microenvironment. Interestingly, Yu and colleagues analyzed the tumor tissue directly by immunofluorescence staining in their nonmyeloablative strategy, applying a low dose (25 mg/kg) of busulfan [96]. In this setup, the tumor-associated myeloid cells could be clearly defined. It was demonstrated that microglia prevailed on days 7 and 17, while comparable numbers of microglia and macrophages were observed on day 14 [96]. Interestingly, chimeras analyzed by flow cytometry revealed divergent results describing either microglia [96] or macrophages [80] dominated tumors. In contrast, in chimeras with high dose (125 mg/kg) of busulfan, around 80% of myeloid cells were infiltrated macrophages [80]. Notably, the high concentration of busulfan led to increased macrophage accumulation in naïve brains, indicating unspecific influx of circulating immune cells under this condition [96].

Another group used the *Cx3cr1^GFP/+^Ccr2^RFP/+^* transgenic mouse strain in combination with two glioma models (PDGFB-driven glioblastoma and GL261) for differentiation [69]. Mice were analyzed at late stages of tumor progression implicating massive glioma growth. Both glioma settings showed similar results. Immunofluorescence analyses revealed predominantly macrophages (GFP^+^RFP^+^) in the tumor area while only a small number of microglia (GFP^+^RFP^-^, 18–24%) was described. Flow cytometry verified these observations, showing that brain tumors were infiltrated by 83% macrophages. Additionally, total body irradiated chimeras were generated and again macrophages represented the main myeloid cell population in glioma tissue [69], as observed previously by using this method [79]. In general, this discrimination was also based on the CD45 expression level and precluded an upregulation of CCR2 on microglia under tumor conditions.

Depending on the method, model and time of glioma progression, microglia or macrophages were postulated to predominate the glioma tissue.

## 6. Conclusions

It is well documented that tumor-associated myeloid cells are highly important for glioma progression. However, past work provides inadequate evidence to finally conclude if discrimination between brain-resident microglia and infiltrating macrophages is required or both populations become same properties following infiltration of the tumor tissue. New reporter mice could help to solve this problem, but many strategies are based on the CD45 expression level. Thus, finding an unequivocal marker, which could be referred to microglia and macrophages, should take priority.

## Figures and Tables

**Table 1:** Contribution of microglia (MG) and macrophages (MO) to glioma tissues.

Model (Tumor Growth)	Method of Distinction	Definition MG/MO	Contribution (%)		Reference
			MG	MO	
rat glioma					
C6 (10–14d) 9L (10–14d) RG-2 (10–14d)	CD45 expression level (FC)	CD45^low^/CD45^high^ of all living cells	26–34 22–24 13–15	9 5–12 3–6	[64]
rat glioma					
C6 (d21)	CD45 expression level (FC)	CD45^low^/CD45^high^ of all living cells	28	3	[66]
murine glioma					
GL261 (d8) GL261 (d15)	CD45 expression level (FC)	CD45^low^/CD45^high^ of the CD11b^+^CD45^+^ cells	83 58	17 42	[144]
murine glioma					
GL261 (d7) GL261 (d14) GL261 (d21)	chimeras: TBI + GFP-BM (FC)		75 68 35	25 32 65	[79]
GL261 (d7) GL261 (d14) GL261 (d21)	chimeras: HPI + GFP-BM (FC)	GFP^-^/GFP^+^ of the CD11b^+^CD45^+^ cells	98 97 75	2 3 25	
murine glioma					
GL261 (d7) GL261 (d14) GL261 (d17)	chimeras: Busulfan (25 mg/kg) + GFP-BM (IF)	IBA1^+^GFP^-^/IBA1^+^GFP^+^ of all IBA1^+^ cells	59 50 56	41 50 44	[96]
GL261 (d17)	chimeras: Busulfan (25 mg/kg) + GFP-BM (FC)	CD45.1^+^/CD45.2^+^ of the CD11b^+^CD45^+^ cells	73	27	
murine glioma					
GL261 (d14)	chimeras: Busulfan (25 mg/kg) + CD45.2-BM (FC)	CD45.1^+^/CD45.2^+^ of the CD11b^+^CD45^+^ cells	33	67	[80]
GL261 (d14)	chimeras: Busulfan (125 mg/kg) + CD45.2-BM (FC)		13	87	
GL261 (d14)	chimeras: HPI + CD45.2-BM (FC)		54	46	
murine glioma					
PDGFB-driven GB (end of life)	CD45 expression level (FC)	CD45^low^/CD45^high^Ly6C^high^, CD45^high^Ly6C^low^ of CD11b^+^CD45^+^ cells	13	83	[69]
PDGFB-driven GB (end of life)	transgenic mice *Cx3cr1^GFP/+^Ccr2^RFP^* (FC)	CX3CR1^high^CCR2^−^/ CX3CR1^+^CCR2^+^	10	82	
PDGFB-driven GB (end of life) GL261 (d21)	transgenic mice *Cx3cr1^GFP/+^Ccr2^RFP^* (IF)	GFP^+^RFP^−^/GFP^+^RFP^+^	24 18	76 82	
GB cells (4–6 weeks)	chimeras: TBI + *Cx3cr1^GFP/+^Ccr2^RFP/+^*-BM (IF)	IBA1^+^GFP^−^/IBA1^+^GFP^+^	17	83	

FC, flow cytometry; IF, immunofluorescence; TBI, total body irradiation; HPI, head-protected irradiation; BM, bone marrow; GB, glioblastoma; d, days after tumor cell implantation.

## Data Availability

Not applicable.

## Abbreviations

BBB	Blood brain barrier
BM	Bone marrow
BMDM	Bone marrow-derived macrophages
CD	Cluster of differentiation
CNS	Central nervous system
EP	Epilepsy patient
FC	Flow cytometry
GB	Glioblastoma
GCV	Ganciclovir
GFP	Green-fluorescent protein
HPI	Head-protected irradiation
IF	Immunofluorescence staining
MG	Microglia
MO	Macrophages
RFP	Red-fluorescent protein
TAMs	Tumor-associated macrophages
TBI	Total body irradiation
WHO	World Health Organization
